# The Association between NICU Admission and Mental Health Diagnoses among Commercially Insured Postpartum Women in the US, 2010–2018

**DOI:** 10.3390/children9101550

**Published:** 2022-10-13

**Authors:** Dana C. Beck, Karen M. Tabb, Anca Tilea, Stephanie V. Hall, Ashlee Vance, Stephen W. Patrick, Amy Schroeder, Kara Zivin

**Affiliations:** 1 School of Nursing, University of California Los Angeles, Los Angeles, CA 90095, USA; 2 School of Social Work, University of Illinois at Urbana-Champaign, Urbana, IL 68101, USA; 3 Department of Obstetrics and Gynecology, University of Michigan, Ann Arbor, MI 48109, USA; 4 Department of Learning Health Sciences, University of Michigan Medical School, Ann Arbor, MI 48109, USA; 5 Department of Psychiatry, University of Michigan Medical School, Ann Arbor, MI 48109, USA; 6 Center for Health Policy and Health Services Research, Henry Ford Health, Detroit, MI 48202, USA; 7 Department of Health Policy, Vanderbilt University Medical Center, Nashville, TN 37232, USA; 8 Vanderbilt Center for Child Health Policy, Vanderbilt University Medical Center, Nashville, TN 37232, USA; 9 Department of Pediatrics, Vanderbilt University Medical Center, Nashville, TN 37232, USA; 10 Mildred Stahlman, Division of Neonatology, Vanderbilt University Medical Center, Nashville, TN 37232, USA; 11 Institute for Healthcare Policy and Innovation, University of Michigan, Ann Arbor, MI 48109, USA

**Keywords:** postpartum, depression, anxiety, NICU, health disparities, maternal health, infant health, dyad, maternal mental health

## Abstract

Maternal mental health (MH) conditions represent a leading cause of preventable maternal death in the US. Neonatal Intensive Care Unit (NICU) hospitalization influences MH symptoms among postpartum women, but a paucity of research uses national samples to explore this relationship. Using national administrative data, we examined the rates of MH diagnoses of anxiety and/or depression among those with and without an infant admitted to a NICU between 2010 and 2018. Using generalized estimating equation models, we explored the relationship between NICU admission and MH diagnoses of anxiety and/or depression, secondarily examining the association of NICU length of stay and race/ethnicity with MH diagnoses of anxiety and/or depression post NICU admission. Women whose infants became hospitalized in the NICU for <2 weeks had 19% higher odds of maternal MH diagnoses (aOR: 1.19, 95% CI: 1.14%–1.24%) and those whose infants became hospitalized for >2 weeks had 37% higher odds of maternal MH diagnoses (aOR: 1.37 95% CI: 1.128%–1.47%) compared to those whose infants did not have a NICU hospitalization. In adjusted analyses, compared to white women, all other race/ethnicities had significantly lower odds of receiving a maternal MH condition diagnosis [Black (aOR = 0.76, 0.73–0.08), Hispanic (aOR = 0.69, 0.67–0.72), and Asian (aOR: 0.32, 0.30–0.34)], despite higher rates of NICU hospitalization. These findings suggest a need to target the NICU to improve maternal MH screening, services, and support while acknowledging the influence of social determinants, including race and ethnicity, on health outcomes.

## 1. Introduction

Maternal mental health (MH) conditions represent a leading cause of preventable maternal mortality in the US [1], implicated in 1 in 9 maternal deaths [2]. Depression and anxiety affect approximately 15% of childbearing women nationally [3,4], and MH symptoms during the perinatal period, such as suicidal ideation and self-harm, have increased over time [5,6]. Traumatic, financial, emotional, or stressful partner-related life events during the antenatal period increase the risk of postpartum depression [7]. Additional risk factors for maternal MH conditions include the absence of social support and trauma histories, including childbirth-related trauma [8,9]. Hospitalization, such as having an infant in the neonatal intensive care unit (NICU), can become a stressful life event and a source of trauma for some [10,11]. Postpartum women who have an infant in NICU care may experience an increased risk of developing MH conditions [10,11,12,13,14,15,16,17,18,19]. However, studies exploring the association of NICU hospitalizations and maternal MH have not used large national samples.

Annually, 1 in 10 families experiences a NICU hospitalization after childbirth in the US [20,21]. NICU admission results from a range of potentially severe infant complications, most commonly preterm birth, low birth weight, or respiratory problems [22]. NICU admissions can serve as a stressful life event for delivering women and for infants, with potential long-term implications for maternal MH and maternal–infant bonding for some families [16,17,18,19]. Research on the psychosocial impact of NICU infant health severity on the family has used length of stay as a metric to explore infant medical complexity [23]. Multiple studies found that a longer length of NICU stay increased the impact on families across a variety of psychosocial dimensions, including increased parental stress and more intrusive parenting styles [23]. A longer duration of NICU hospitalization increases emotional distress among postpartum women [16,17,18]. In the NICU, the focus is on infant health; therefore, maternal MH disturbances associated with circumstantial stress related to NICU hospitalization remain underrecognized [24]. Addressing maternal MH in the context of the NICU could have important implications for supporting the health of the family unit to better meet the needs of the infant.

Limited literature evaluates the relationship between NICU hospitalization and symptoms of depression and anxiety among postpartum women. In addition, the existing literature uses cross-sectional designs [12,13,14,17,18], small sample sizes [12,13,14,17,18,19], a focus on a single region [12,13,14,15,16,17,18], and qualitative methodologies [15]. Additionally, it does not typically control for antenatal MH conditions [10,17,18]. Furthermore, there is a need to explore whether social determinants of health such as race/ethnicity retain an association with maternal MH after controlling for NICU experiences, as families who are of non-white race can experience neglectful or judgmental care and worse infant health outcomes [25,26,27].

A scoping review examining the MH of parents with infants in the NICU found that MH concerns are common in parents of NICU infants, but studies in the US primarily included white participants or did not include the race/ethnicity of participants [28]. In cross-sectional studies using small (N = 40–200) convenience samples at single centers among predominantly white women with recent infant NICU experiences, 21%–43% experienced postpartum depression or anxiety symptoms using the Edinburgh Postnatal Depression [EPDS] or Anxiety [EPDS-A] Scales [12,13,29]. Among postpartum women in California, Chan et al. (2021) found non-Hispanic Black postpartum women were more likely to receive MH care in inpatient psychiatric settings compared to non-Hispanic white, Hispanic, non-Hispanic Asian, and other race/ethnicities, suggesting a potential failure of early diagnosis and treatment of MH needs among non-Hispanic Black postpartum women [30]. Indeed, a single state retrospective cohort study found that Black childbearing women were less likely to be screened for MH conditions compared to women of other racial and ethnic makeup [31]. Further, a recent study in California showed non-Latina Black and non-Latina Asian postpartum women with postpartum MH symptoms had significantly lower odds of receiving MH treatment compared to white women [32]. Disparities in maternal MH identification and treatment persist, as minoritized women may have a higher prevalence of maternal MH conditions [33,34] and lower odds of receiving diagnosis and treatment [34,35]. Yet, to our knowledge, there are no nationally representative studies that explore the relationship between race/ethnicity and postpartum mental health [36].

Thus, our study aimed to expand upon the existing literature by exploring diagnoses of maternal MH conditions (anxiety and depression) among postpartum women with an infant admitted to the NICU compared to those without NICU admission in a national commercially insured sample. Further, we sought to identify whether social determinants, such as race/ethnicity (Black, Hispanic, and Asian versus white), retained an association with maternal MH after controlling for NICU experiences.

## 2. Materials and Methods

**Figure 1:**
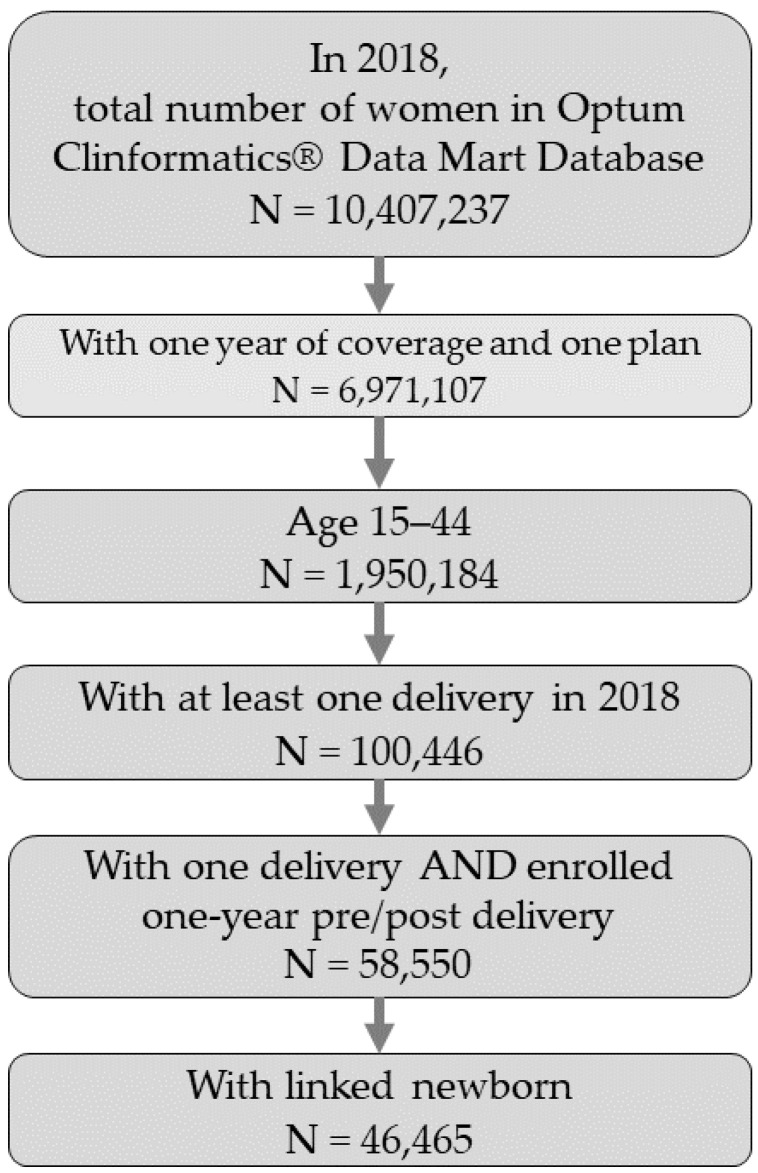
Cohort construction flowchart of inclusion and exclusion criteria, using 2018 as an example.

This retrospective cohort study evaluated the prevalence of maternal MH condition diagnoses of anxiety and/or depression identified within the year following birth among women aged 15–44 with and without an infant admitted to the NICU using Optum’s deidentified Clinformatics^®^ Data Mart Database (CDM). CDM includes a statistically deidentified large claims data warehouse of administrative health claims from all 50 states. We identified postpartum women from 2010 to 2018 and only included those who had continuous enrollment in a single employer-based health plan for at least 1 year before and 1 year after a live birth. For these women, using a family identifier variable, we linked family members, and using year of birth, we identified newborns within a family. We restricted our analytical cohort to postpartum women with linked newborns (Figure 1).

We identified women whose infants had a NICU admission (CPT codes 99468, 99469, 99477, 99478, 99479, 99480) and who had MH diagnoses of anxiety and/or depression (see Appendix A, Appendix B and Appendix C) up to one year or anytime in the year following delivery using standardized International Classification of Disease, 9th and 10th Revision, Clinical Modification diagnosis codes present at least once in inpatient claims or twice in outpatient claims. We selected sociodemographic covariates based upon factors established to influence the relationship between NICU hospitalization and maternal MH [10,11,12,13,14,16,17,18,19]. Covariates included age (≤18, 19–26, 27–34, 35–39, ≥40), race/ethnicity (Asian, Black, Hispanic, Unknown race/ethnicity, white), region (Midwest, West, Northeast, South), insurance type (Point of Service, Exclusive Provider Organization/Health Maintenance Organization, Preferred Provider Organization, other), other MH conditions, and substance use disorders. We identified other MH conditions not related to anxiety or depression, such as bipolar disorder, schizophrenia, and other conditions, as well as substance use disorder conditions such as alcohol, tobacco, cannabis, and other conditions using ICD-9 and ICD-10 codes (see Appendix B and Appendix C, respectively). We used a similar algorithm of one inpatient or two outpatient claims to assess the prevalence of other MH conditions or substance use disorders. The University of Michigan Institutional Review Board (HUM00188304) approved this study.

### Statistical Analysis

Using means or proportions with the associated 95% confidence intervals we evaluated demographic and clinical characteristics including age, race/ethnicity, region, insurance type, other MH conditions, and substance use disorders for all women, and for postpartum women whose infants had a NICU admission for <14 days or ≥14 days for the years between 2010 and 2018. Using all study period data (2010–2018), we examined trends in NICU admission rates, overall, by maternal MH status and by postpartum individual’s race/ethnicity. NICU admission was the primary predictor of interest and maternal MH diagnoses of anxiety and/or depression the primary outcome; secondarily, we explored maternal MH by NICU length of stay and race/ethnicity. We used generalized estimating equation (GEE) models with an exchangeable covariance structure to control for repeating deliveries (women who gave birth more than once during the years 2010–2018) to explore the association between maternal MH diagnoses of anxiety and/or depression and NICU admission adjusting for the following covariates chosen a priori: delivery year, postpartum individual’s age, race/ethnicity, insurance plan type, region, other MH conditions, and substance use disorders. We conducted all analyses and data management using SAS v9.4 (Cary, NC, USA).

## 3. Results

Our study cohort included 533,080 delivering women, for whom we identified 446,553 newborns (83.7%) between 2010 and 2018. Of the 55,500 women who delivered in 2010, 8.8% (95% CI: 8.6%–9.0%) had newborns with NICU admission and 11.9% (95% CI: 11.6%–12.1%) received a MH diagnosis of anxiety and/or depression in the postpartum period (Table 1a). Of the 49,431 women who delivered in 2018, 10.4% (95% CI: 10.1%–10.6%) had newborns with NICU admission and 18.1% (95% CI: 17.7%–18.4%) received a MH diagnosis of anxiety and/or depression in the postpartum period (Table 1b).

**Figure 2:**
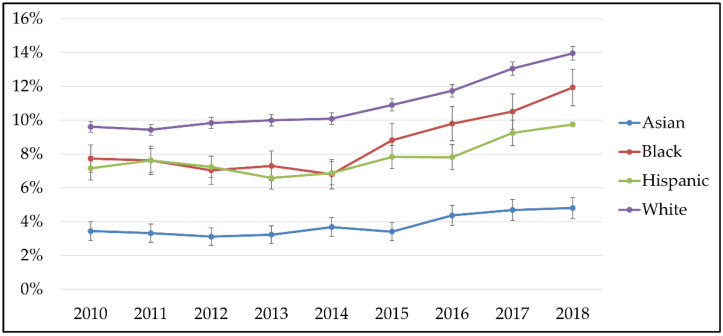
Proportion of postpartum anxiety or depression, by race/ethnicity, 2010–2018.

The proportion of postpartum women diagnosed with anxiety and/or depression among women without prenatal diagnoses of anxiety or depression increased over time overall and for all race/ethnicities (Figure 2): 9.6% (95% CI: 9.3%–9.9%) of white postpartum women had an MH diagnosis of anxiety and/or depression in 2010 and 13.9% (95% CI: 13.5%–14.4%) in 2018; 3.4% (95% CI: 2.9%–4.0%) of Asian women had a diagnosis of postpartum anxiety and/or depression in 2010 and 4.8% (95% CI: 4.2%–5.4%) in 2018; 7.2% (95% CI: 6.5%–7.8%) of Hispanic women had a postpartum diagnosis of anxiety and/or depression in 2010 and 9.8% (95% CI: 9.0%–10.5%) in 2018; and 7.7% (95% CI: 6.9%–8.5%) of Black women had a diagnosis of postpartum anxiety and/or depression in 2010 and 11.9% (5% CI: 10.9%–13.0%) in 2018.

**Figure 3:**
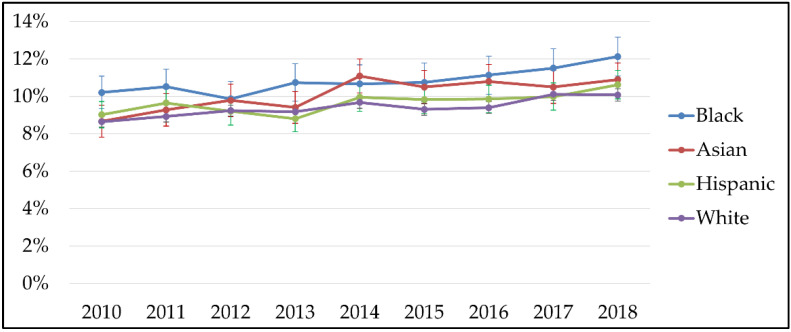
Proportion of NICU newborns, by race/ethnicity, 2010–2018.

Between 2010 and 2018, rates of NICU admission increased among all infants, regardless of race/ethnicity, including: Black infants from 10.2% (95% CI: 9.3%–11.1%) to 12.1% (95% CI: 11.1%–13.2%); Asian infants from 8.7% (95% CI: 7.8%–9.5%) to 10.9% (95% CI: 10.0%–11.8%); Hispanic infants from 9.0% (95% CI: 8.3%–9.7%) to 10.6% (95% CI: 9.9%–11.4%); and white infants from 8.6% (95% CI: 8.3%–8.9%) to 10.0% (95% CI: 9.7%–10.4%; Figure 3).

After adjusting for delivery year, age, race/ethnicity, region, insurance, other MH conditions, and substance use disorders, postpartum women whose infants experienced NICU admission had 23% higher odds (aOR: 1.23, 95% CI: 1.19%–1.27%) of an MH diagnosis of anxiety and/or depression than those women without NICU admission (Table 2a). Furthermore, after restricting the analysis to women who did not have a MH diagnosis in the prenatal period, for women whose infant’s NICU experience lasted less than two weeks, we observed a 19% increase in odds of postpartum MH condition diagnoses of anxiety and/or depression (aOR: 1.19, 95% CI: 1.14%–1.24%; Table 2b) compared to women with no NICU admission. For women whose infant’s length of stay exceeded two weeks, we observed a 37% increase in odds of postpartum anxiety and/or depression (aOR: 1.37, 95% CI: 1.13%–1.47%) compared to women who did not have an infant with a NICU admission (Table 2b).

In adjusted analyses, compared to white women, other race/ethnicities had lower odds of a postpartum MH condition diagnosis of anxiety and/or depression: Black (aOR: 0.76, 95% CI: 0.74%–0.79%), Hispanic (aOR: 0.69, 95% CI: 0.66%–0.71%), and Asian (aOR: 0.35, 95% CI: 0.34%–0.37%), shown in Table 2b. This trend of lower odds of a postpartum MH condition diagnosis in racial/ethnic minorities was similar when the analyses were not restricted to women who did not have a MH diagnosis in the prenatal period.

## 4. Discussion

This multi-year study of a commercially insured US population found that postpartum women whose infants had NICU hospitalization had higher odds of an anxiety and/or depression diagnosis in the year following this experience compared to those who did not have an infant hospitalized in the NICU. Trends in NICU admission increased slightly during the study period, consistent with national averages of NICU stays [21]. Incidence of postpartum MH diagnoses of anxiety and/or depression increased during the study period. Odds of MH diagnoses of anxiety and/or depression increased as the length of stay in the NICU increased. These findings echo results of smaller studies that found NICU admission influenced MH symptoms of depression and anxiety among delivering women [10,11,12,13,14,15,16,17,18,19]. Our findings extend the results of these smaller studies expanding the sample size, region, and racial/ethnic diversity of women included for analysis in NICUs in the US. Through conducting an additional analysis that only includes postpartum women without prenatal MH conditions, we further confirm the relationship between NICU admission and incidence of MH diagnoses of anxiety and/or depression among postpartum women.

Longer length of NICU stay translated to higher risk for postpartum MH diagnoses of anxiety and/or depression in the sample of women without prenatal MH conditions. Postpartum women with infants in the NICU for less than two weeks had increased odds of MH diagnoses of anxiety and/or depression. These odds nearly doubled when NICU length of stay exceeded two weeks. Our study found that any NICU hospitalization increased postpartum MH diagnoses, but given the range of infant health severity in the NICU, a longer infant length of stay appears to be associated with a higher risk of maternal MH conditions. Using NICU resources to screen for and address the MH of postpartum women may prove beneficial, and women with infants in the NICU for prolonged periods of time may have an increased need for support [24]. Several studies indicate the benefit of individualized interventions using trauma-informed modalities to address the health of infants and their parents [37].

We found a higher proportion of Black, Hispanic, and Asian infants with NICU admission than white infants over time in this sample, mirroring other national research [21,30,31,32,33,34,35]. Although Black, Asian, and Hispanic infants had higher rates of NICU admission between 2010 and 2018, white postpartum women had higher rates of MH diagnoses of anxiety and/or depression compared to Black, Asian, or Hispanic women. Further, NICU admission increased the risk for these postpartum MH diagnoses, but prevalence of NICU admission by race/ethnicity did not correspond with diagnosis of postpartum MH conditions by race/ethnicity. Black, Hispanic, and Asian women had a higher proportion of infants in the NICU, yet a higher proportion of white postpartum women received maternal MH diagnoses, in accordance with similar research [30,31,32,33,34,35]. This counterintuitive finding may reflect the reality of experienced MH conditions and that white women do indeed experience worse mental health after childbirth. However, the surrounding body of research in this area supports the interpretation that this may describe evidence of a racial disparity in MH diagnoses for women with infants in the NICU [30,31,32,33,34,35]. Thus, another way to interpret our findings is that Black, Hispanic, and Asian women had lower odds of receiving a postpartum MH diagnosis compared to white women, and that may not describe the actual prevalence of these conditions among these racial/ethnic populations.

Although much of the NICU admission rightly focuses on the health of the infant, our study calls for attention to the well-being of postpartum women. Prior literature provides detailed guidance on the best practices for detecting MH conditions among all delivering women with infants hospitalized in the NICU, suggesting that care for parents’ emotional well-being in the NICU represents an important component to the care of their hospitalized infants [24]. Considering care of the dyad, including both clinical and psychosocial needs, remains integral to family health and well-being; multidisciplinary care in the NICU environment can address these care gaps [24]. The 4th Trimester Project (North Carolina) [38] and Firefly (Tennessee) [39] promote patient-centered care among under-resourced women during the postpartum period. These state efforts are improving the health and well-being for postpartum women, their infants, and families. Implementation of comprehensive interventions at the state and community level could tailor to the unique needs of under-resourced communities nationally to improve quality of care in the NICU and into the first years of life for infants and families.

This study has multiple strengths, including documentation of trends in NICU admission and postpartum MH diagnoses of anxiety and/or depression over time using a large, national sample and observation of dyadic outcomes. Along with these strengths, this work also has limitations. First, we used a privately-insured sample. Thus, these data do not reflect dyadic outcomes among nearly half the births in the US, which have public insurance coverage or remain uninsured, in the postpartum period. This sample selection represents one possible explanation for the racially and ethnically disparate findings of this study. Second, this study used ICD codes. Therefore, these data could not reliably indicate screening rates for maternal MH conditions and elevated symptoms for MH problems, a limitation which could have implications for potential racial/ethnic disparities. Since there are studies that document racial/ethnic disparities in screening rates and inadequate diagnoses/treatment of mental health conditions for racial/ethnic minorities compared to white people, our study may have not captured the accurate association between race/ethnicity and postpartum mental health. Third, this study could not address the mechanisms driving the differential rates of MH diagnosis across racial and ethnic groups of women with an infant in the NICU.

Some evidence suggests that legislating screening for maternal MH conditions may reduce inequities associated with screening and improving screening rates [32]. Yet, few postpartum women receive screening, diagnosis, follow-up, and adequate treatment, in part, because of uncoordinated systems of care across clinical specialties [40], with glaring disparities documented by race/ethnicity [33,34,35]. Addressing disparities in comprehensive MH care among postpartum women requires coordinated care across specialty inpatient settings, such as NICU, and extending into ambulatory care settings [24]. Improving care for dyads in the NICU and beyond also requires acknowledging the role that systemic factors, including social determinants of health, play in disparate MH care seeking, delivery, and outcomes [41].

In conclusion, this study found that NICU hospitalization increases the odds of maternal MH diagnoses of anxiety and/or depression in the year following this event, with longer length of infant hospitalization contributing to significantly higher rates of these maternal MH diagnoses. Although Black, Asian, and Hispanic infants had higher rates of NICU hospitalization over time, white postpartum women had higher rates of MH diagnoses. The interdisciplinary nature of the NICU provides an excellent opportunity to further investigate the social determinants of health and promote maternal-infant health.

## Figures and Tables

**Table 1:** (**a**) Cohort characteristics, in 2010, overall and by NICU status. (**b**) Cohort characteristics, in 2018, overall and by NICU status.

**(a)**								
**Demographic**	**Overall**	**Overall %**	**No NICU**	**No NICU % 95% CI**	**<14 days NICU**	**<14 days NICU % 95% CI**	**≥14 days NICU**	**≥14 days NICU %**
		**95% CI**						**95% CI**
	55,500		50,614		3671		1215	
**Age**								
≤18	220	0.40%	210	95.45% [92.64, 98.27]	(<11)	3.18%	(<11)	1.36%
		[−0.43, 1.23]				[−9.82, 16.18]		[−11.8, 14.49]
19–26	6689	12.05% [11.27, 12.83]	6131	91.66% [90.97, 92.35]	436	6.52% [4.20, 8.84]	122	1.82%
								[−0.55, 4.20]
27–34	32,663	58.85% [58.32, 59.39]	29,944	91.68% [91.36, 91.99]	2047	6.27% [5.22, 7.32]	672	2.06%
								[0.98, 3.13]
35–39	12,388	22.32% [21.59, 23.05]	11,211	90.50% [89.96, 91.04]	876	7.07% [5.37, 8.77]	301	2.43%
								[0.69, 4.17]
≥40	3540	6.38% [5.57, 7.18]	3118	88.08% [86.94, 89.22]	305	8.62% [5.47, 11.76]	117	3.31%
								[0.07, 6.54]
**Race/Ethnicity**								
Asian	4236	7.63% [6.83, 8.43]	3869	91.34% [90.45, 92.22]	277	6.54% [3.63, 9.45]	90	2.12%
								[−0.85, 5.10]
Black	4692	8.45% [7.66, 9.25]	4213	89.79% [88.88, 90.71]	335	7.14% [4.38, 9.90]	144	3.07%
								[0.25, 5.89]
Hispanic	6353	11.45% [10.66, 12.23]	5780	90.98% [90.24, 91.72]	438	6.89% [4.52, 9.27]	135	2.12%
								[−0.31, 4.56]
Unknown race	4149	7.48% [6.68, 8.28]	(<11)		(<11)		(<11)	
White	36,070	64.99% [64.50, 65.48]	32,954	91.36% [91.06, 91.66]	2346	6.50% [5.51, 7.50]	770	2.13%
								[1.11, 3.16]
**Region**								
Midwest	12,990	23.41% [22.68, 24.13]	11,913	91.71% [91.21, 92.20]	811	6.24% [4.58, 7.91]	266	2.05%
								[0.35, 3.75]
West	11,059	19.93% [19.18, 20.67]	10,185	92.10% [91.57, 92.62]	663	6.00% [4.19, 7.80]	211	1.91%
								[0.06, 3.75]
Northeast	6067	10.93% [10.15, 11.72]	5466	90.09% [89.30, 90.89]	479	7.90% [5.48, 10.31]	122	2.01%
								[−0.48, 4.50]
South	25,350	45.68% [45.06, 46.29]	23,017	90.80% [90.42, 91.17]	1717	6.77% [5.58, 7.96]	616	2.43%
								[1.21, 3.65]
**Insurance**								
POS	38,661	69.66% [69.20, 70.12]	35,218	91.09% [90.80, 91.39]	2583	6.68% [5.72, 7.64]	860	2.22%
								[1.24, 3.21]
EPO/HMO	15,656	28.21% [27.50, 28.91]	14,304	91.36%	1011	6.46% [4.94, 7.97]	341	2.18%
				[90.90, 91.82]				[0.63, 3.73]
PPO	983	1.77% [0.95, 2.60]	905	92.07%	65	6.61% [0.57, 12.65]	13	1.32%
				[90.30, 93.83]				[−4.89, 7.53]
Other	200		187	93.50%	12	6.00%	(<11)	0.50%
				[89.97, 97.03]		[−7.44, 19.44]		[−13.3, 14.32]
**Other MH Conditions (excluding anxiety and depression)**								
No other MH	52,450	94.50% [94.31, 94.70]	47,892	91.31% [91.06, 91.56]	3420	6.52% [5.69, 7.35]	1138	2.17%
								[1.32, 3.02]
Pre-other MH	2983	5.37% [4.57, 6.18]	2657	89.07% [87.89, 90.26]	249	8.35% [4.91, 11.78]	77	2.58%
								[−0.96, 6.12]
**Substance**								
**Use Disorder**								
No SUD	54,751	98.65% [98.55, 98.75]	49,973	91.27% [91.03, 91.52]	3596	6.57% [5.76, 7.38]	1182	2.16%
								[1.33, 2.99]
Pre-SUD	749	1.35% [0.52, 2.18]	641	85.58% [82.86, 88.30]	75	10.01% [3.22, 16.81]	33	4.41%
								[−2.60, 11.41]
**(b)**								
**Demographic**	**Overall**	**Overall %**	**No NICU**	**No NICU % 95% CI**	**<14 days NICU**	**<14 days NICU % 95% CI**	**≥14 days NICU**	**≥14 days NICU %**
		**95% CI**						**95% CI**
	49,431		44,301		3986		1144	
**Age**								
≤18	57		50	87.72% [78.62, 96.82]	(<11)	10.53%	(<11)	1.75%
						[−14.0, 35.08]		[−24.0, 27.49]
19–26	3722	7.53% [6.68, 8.38]	3388	91.03% [90.06,91.99]	274	7.36% [4.27,10.45]	60	1.61%
								[−1.57, 4.80]
27–34	29,059	58.79% [58.22, 59.35]	26,212	90.20% [89.84, 90.56]	2224	7.65% [6.55, 8.76]	623	2.14%
								[1.01,3.28]
35–39	13,455	27.22% [26.47, 27.97]	11,985	89.07% [88.52, 89.63]	1147	8.52% [6.91, 10.14]	323	2.40%
								[0.73, 4.07]
≥40	3138	6.35% [5.50, 7.20]	2666	84.96% [83.60, 86.32]	335	10.68% [7.37, 13.98]	137	4.37%
								[0.94, 7.79]
**Race/Ethnicity**								
Asian	4635	9.38% [8.54, 10.22]	4130	89.10% [88.15, 90.05]	394	8.50% [5.75, 11.25]	111	2.39%
								[−0.45, 5.24]
Black	3834	7.76% [6.91, 8.60]	3369	87.87% [86.77, 88.97]	349	9.10% [6.08, 12.12]	116	3.03%
								[−0.09, 6.14]
Hispanic	6458	13.06% [12.24, 13.89]	5772	89.38% [88.58, 90.17]	533	8.25% [5.92, 10.59]	153	2.37%
								[−0.04, 4.78]
Unknown race	2429	4.91% [4.05, 5.77]	(<11)		(<11)		(<11)	
White	32075	64.89% [64.37, 65.41]	28843	89.92% [89.58, 90.27]	2521	7.86% [6.81, 8.91]	711	2.22%
								[1.13, 3.30]
**Region**								
Midwest	13,224	26.75% [26.00, 27.51]	11,858	89.67% [89.12, 90.22]	1049	7.93% [6.30, 9.57]	317	2.40%
								[0.71, 4.08]
West	11,018	22.29% [21.51, 23.07]	9923	90.06% [89.47, 90.65]	838	7.61% [5.81, 9.40]	257	2.33%
								[0.49, 4.18]
Northeast	5275	10.67% [9.84, 11.50]	4659	88.32% [87.40, 89.24]	515	9.76% [7.20, 12.33]	101	1.91%
								[−0.76, 4.59]
South	19,738	39.93% [39.25, 40.61]	17,700	89.67% [89.23, 90.12]	1572	7.96% [6.63, 9.30]	466	2.36%
								[0.98, 3.74]
**Insurance**								
POS	37,235	75.33% [74.89, 75.77]	33,386	89.66% [89.34, 89.99]	2983	8.01% [7.04, 8.99]	866	2.33%
								[1.32, 3.33]
EPO/HMO	11,070	22.39% [21.62, 23.17]	9893	89.37% [88.76, 89.98]	918	8.29% [6.51, 10.08]	259	2.34%
								[0.50, 4.18]
PPO	630	1.27% [0.40, 2.15]	576	91.43% [89.14, 93.71]	45	7.14%	(<11)	1.43%
						[−0.38, 14.67]		[−6.32, 9.18]
Other	496	1.00% [0.13, 1.88]	446	89.92% [87.13, 92.71]	40	8.06%	(<11)	2.02%
						[−0.37, 16.50]		[−6.70, 10.73]
**Other MH Conditions (excluding anxiety and depression)**								
No other MH	42,773	86.53% [86.21, 86.85]	38,499	90.01% [89.71, 90.31]	3336	7.80% [6.89, 8.71]	938	2.19%
								[1.26, 3.13]
Pre-other MH	6518	13.19% [12.36, 14.01]	5673	87.04% [86.16, 87.91]	645	9.90% [7.59, 12.20]	200	3.07%
								[0.68, 5.46]
**Substance Use Disorder**								
No SUD	47,883	96.87% [96.71, 97.02]	43,013	89.83% [89.54, 90.12]	3783	7.90% [7.04, 8.76]	1087	2.27%
								[1.38, 3.16]
Pre-SUD	1548	3.13% [2.26, 4.00]	1288	83.20% [81.16, 85.25]	203	13.11% [8.47, 17.76]	57	3.68%
								[−1.21, 8.57]

Abbreviations: Neonatal Intensive Care Unit = NICU; Point of Service = POS; Exclusive Provider Organization/Health Maintenance Organization = EPO, HMO; Preferred Provider Organization = PPO; Mental health = MH; Substance use disorder = SUD. For cell counts < 11, data was removed.

**Table 2:** (**a**) Adjusted odds ratio and confidence intervals for predicting postpartum MH diagnoses of anxiety and/or depression. NICU categories include no NICU and yes NICU. (**b**) Adjusted odds ratio and confidence intervals for predicting postpartum MH diagnoses of anxiety and/or depression among women with no prenatal MH diagnosis. NICU categories include no NICU, <14 days, and ≥14 days.

**(a)**				
**Covariate**	**OR**	**95%CI**		***p*-Value**
Delivery Year	1.057	1.053	1.062	<0.0001
**NICU vs. no NICU**	1.234	1.192	1.278	<0.0001
**Race/Ethnicity (ref: White)**				
Asian	0.357	0.340	0.375	<0.0001
Black	0.769	0.741	0.799	<0.0001
Hispanic	0.690	0.668	0.713	<0.0001
**Age (ref: ≤18)**				
19–26	1.066	0.870	1.306	0.5369
27–34	1.018	0.833	1.245	0.8605
35–39	1.080	0.883	1.321	0.4548
≥40	1.141	0.930	1.399	0.2051
**Insurance (ref: HMO/EPO)**				
POS	1.004	0.980	1.028	0.7485
PPO	1.129	1.038	1.227	0.0045
Other	1.059	0.944	1.188	0.3265
**Region (ref: West)**				
Midwest	1.142	1.109	1.175	<0.0001
Northeast	1.018	0.981	1.056	0.3414
South	1.010	0.983	1.038	0.4511
No SUD vs. Yes SUD	0.572	0.540	0.605	<0.0001
No Other MH vs. Yes Other MH	0.535	0.518	0.552	<0.0001
No pre-MH diagnoses of anxiety and/or depression vs. yes pre-MH diagnoses of anxiety and/or depression	0.145	0.141	0.149	<0.0001
(**b**)				
**Covariate**	**OR**	**95%CI**		***p*-Value**
Delivery Year	1.057	1.053	1.062	<0.0001
**NICU status (ref: No NICU)**				
NICU <14 days	1.194	1.149	1.241	<0.0001
NICU ≥14 days	1.375	1.285	1.472	<0.0001
**Race/Ethnicity (ref: White)**				
Asian	0.324	0.306	0.343	<0.0001
Black	0.768	0.735	0.803	<0.0001
Hispanic	0.697	0.672	0.724	<0.0001
**Age (ref: ≤18)**				
19–26	0.947	0.760	1.181	0.6305
27–34	0.867	0.697	1.079	0.2015
35–39	0.901	0.723	1.121	0.3497
≥40	0.934	0.747	1.166	0.5451
**Insurance (ref: HMO/EPO)**				
POS	0.993	0.966	1.021	0.6274
PPO	1.083	0.979	1.197	0.1214
Other	1.005	0.873	1.156	0.9499
**Region (ref: West)**				
Midwest	1.102	1.067	1.140	<0.0001
Northeast	0.935	0.895	0.977	0.0027
South	0.990	0.960	1.021	0.5355
No SUD vs. Yes SUD	0.545	0.507	0.585	<0.0001
No Other MH vs. Yes Other MH	0.454	0.437	0.472	<0.0001

Abbreviations: Neonatal Intensive Care Unit = NICU; Point of Service = POS; Exclusive Provider Organization/Health Maintenance Organization = EPO, HMO; Preferred Provider Organization = PPO; Mental health = MH; Substance use disorder = SUD.

## Data Availability

The data presented in this study were utilized under a data use agreement between the University of Michigan and Optum and thus are not publicly available.

## Appendix A. ICD-9 and ICD-10 Codes Used to Identify Maternal MH Disorders

**Group**	**ICD Diagnosis Code**
Anxiety	300.xx, 308.xx, 313.xx, 293.xx, F06.xx, F40.xx, F41.xx, F42.xx, F43.xx, F48.xx, R45.xx
Depression	311.xx, 296.xx, 300.xx, F32.xx, F33.xx

## Appendix B. ICD-9 and ICD-10 Codes Used to Identify Other MH Disorders

**Group**	**ICD Diagnosis Code**
Other MH Disorders	312.xx, 314.xx, 313.xx, F90.xx, F91.xx, R46.xx, R46.xx, R46.xx, R46.xx, 309.xx, 309.xx, F43.xx

## Appendix C. ICD and CPT/HCPCS Codes Used to Identify Substance Use Disorder

**Group**	**ICD Diagnosis Code**
ICD Diagnoses	291.xx, 357.xx, 425.xx, 535.xx, 571.xx, 980.xx, 303.xx, 305.xx, 760.xx, F10.xx, G62.xx, I42.xx, K29.xx, K70.xx, O99.xx, 304.xx, F12.xx, F14.xx, 292.xx, 779.xx, 648.xx, 655.xx, 965.xx, F55.xx, O35.xx, F16.xx, F18.xxP04.xx, P96.xx, Q86.xx, F11.xx, F15.xx, F19.xx, F13.xx, T36.xx, T37.xx, T38.xx, T39.xx, T40.xx, T41.xx, T42.xx, T43.xx, T44.xx, T45.xx, T46.xx, T47.xx, T48.xx, T49.xx, T50.xx, T51.xx, T52.xx, T53.xx, T54xx, T55.xx, T56.xx, T57.xx, T58.xx, T59.xx, T60.xx, T61.xx, T62.xx, T63.xx, T64.xx, T65.xx, T71.xx, V65.xx, F17.xx
CPT/HCPCS	G0397, G0396, H0048, H2034, H0014, H0027, H0029, H0007, H0016, H0026, H0028, H0003, H0009, H0005, H0015, H001, H0012, H0011, H0013, H0020, H0008, T1006, H0050, H0022, H0006, H0021, H0047, H2036, H2035, H0001, H0049, T102, T1007, T1011, S9475, G0442, H0039, H0040, H0004, H2012, H0030, H0023, H0025, H0017, H0024, H0002, H0019, H018, G0443, T1009, H2015, H2016, H2011, T1008, T1010, H2025, H2026, H0033, H2001, H0038, H2014, H2019, H2020, H2ZZZZ, HZ63ZZZ, HZ33ZZZ, GZ3ZZZZ, HZ82ZZZ, HZ83ZZZ, HZ87ZZZ, HZ86ZZZ, HZ81ZZZ, HZ85ZZZ, HZ84ZZZ, HZ80ZZZ, HZ89ZZZ, HZ88ZZZ, HZ93ZZZ, HZ97ZZZ, HZ96ZZZ, HZ92ZZZ, HZ95ZZZ, HZ94ZZZ, HZ98ZZZ, HZ91ZZZ, HZ90ZZZ, HZ99ZZ
ICD Procedures	9462, 9461, 9463, 946, 9446, 9469, 9468, 9467, 9465, 9464, 9466
